# Fabrication of Wearable Transistor with All-Graphene Electrodes via Hot Pressing

**DOI:** 10.3390/polym14132602

**Published:** 2022-06-27

**Authors:** Youn Kim, Jin-Yong Hong, Young-Pyo Jeon, Jung Bin Park, Cheol Jin Lee, Jea Uk Lee

**Affiliations:** 1 School of Electrical Engineering, Korea University, Seoul 02841, Korea; younkim@emaxsol.com; 2 Carbon Industry Frontier Research Center, Korea Research Institute of Chemical Technology (KRICT), 141 Gajeong-ro, Yuseong-gu, Daejeon 34114, Korea; jyhong@krict.re.kr (J.-Y.H.); ypjeon@krict.re.kr (Y.-P.J.); 3 Department of Advanced Materials Engineering for Information and Electronics, Integrated Education Institute for Frontier Science and Technology (BK21 Four), Kyung Hee University, 1732 Deogyeong-daero, Giheung-gu, Yongin-si 17104, Gyeonggi-do, Korea; bin01@khu.ac.kr

**Keywords:** textile, graphene, electrode, hot pressing, transistor

## Abstract

Textile electronics are ideal for novel electronic devices owing to their flexibility, light weight, and wearability. In this work, wearable organic field-effect transistors (OFETs) with all-graphene electrodes, fabricated using hot pressing, are described. First, highly conductive and flexible electrodes consisting of a cotton textile substrate and electrochemically exfoliated graphene (EEG) were prepared via hot pressing. The EEG/textile electrodes exhibited a low sheet resistance of 1.3 Ω sq^−1^ and high flexibility; these were used as gate electrodes in the wearable OFETs. In addition, spray-coated EEG was also used as the source/drain (S/D) electrodes of the wearable OFETs, which recorded a sheet resistance of 14.8 Ω sq^−1^ after hot pressing. The wearable OFETs exhibited stable electrical performance, a field-effect mobility of 13.8 cm^2^ V^−1^ s^−1^, and an on–off current ratio of ~10^3^ during 1000 cycles of bending. Consequently, the fabrication method for wearable transistors developed using textiles and hot-pressed graphene electrodes has potential applications in next-generation wearable devices.

## 1. Introduction

The development of advanced and ultra-flexible electronic devices has urged the creation of new types of electronic units and components. Textile electronics, where electronic components and functions are integrated into textile substrates, have attracted much scientific attention because they provide lighter weight and greater flexibility, functionality, and wearability than conventional electronic devices. It is expected that textile electronics will be applied in various fields, such as sensor, display, military, and energy applications [1,2,3].

**Figure 1:**
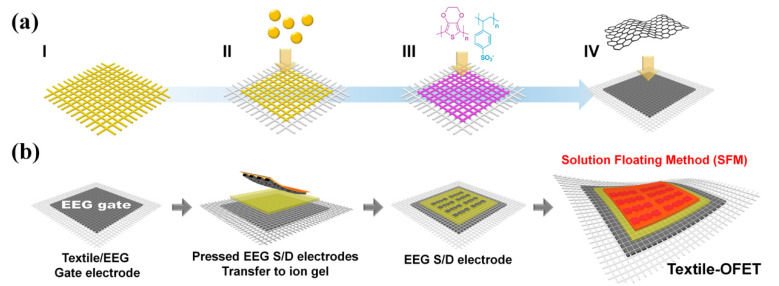
(**a**) Schematic illustration of four methods to produce textile-based electrodes for wearable transistors: (I) utilizing metal wire and mesh, (II) coating metal particles onto textile, (III) conducting polymer coating, and (IV) nano-carbon coating. (**b**) Schematic illustration of the fabrication of a textile-OFET using hot-pressed EEG electrodes.

Currently, some studies have developed textile-based flexible transistors, which are the most important parts in wearable electronic devices such as wearable displays, biomedical sensors, and cloth-based computers [4,5]. There are four pathways to produce textile-based electrodes for a wearable transistor (Figure 1a). Utilizing metal wire and mesh (I) for the gate electrode is convenient for developing the transistor components on top, owing to their high electrical conductivity and rigid form [6]. However, metal-based textile transistors are not suitable for use in wearable electronics because they lack flexibility and human compatibility. Coating the textile with metal particles (II) is very common but difficult for mass production, because the evaporation and deposition of metal atoms need special equipment, which is not economical for industrialization [7]. In addition, the high density of metal and the difficulty in processing metals onto thin textile substrates limit the applications for advanced mobile and wearable applications. Conducting polymer coating (III) is also not practical for wearable transistor applications, owing to the relatively low electrical conductivity and poor environmental stability of polymer materials [8].

A nano-carbon coating, especially using two-dimensional (2D) graphene sheets (IV), can address the above concerns owing to its high electrical conductivity, flexibility, and structural stability [9,10,11]. Many research groups have prepared graphene using different synthetic methods and have already used it as an electrode for electronic devices with a hard substrate [12]. Ahn et al. reported that chemical vapor deposition (CVD) graphene can be assembled onto flexible or stretchable substrates under ambient conditions to manufacture field-effect transistors (FETs) and logic circuits [13]. However, devices formed on porous and 3D structural substrates such as rubber and textiles, with very thin CVD graphene electrodes, may face severe degradation of their electrical performances due to the physical damage of the CVD graphene layers. Our group previously reported the preparation of a conductive textile-based electrode by vacuum filtration and wet transfer of graphene oxide (GO), in association with metal nanoparticle embedding [14]. Although chemically synthesized graphene (GO and reduced graphene oxide (rGO)) have excellent dispersity and processability, they exhibit limitations in their electrical properties for the electrode applications of textile electronics. Therefore, in order to realize truly wearable transistor devices, it is necessary to develop a flexible and robust graphene layer with high conductivity on the textile substrate.

In this work, highly conductive and flexible electrodes based on electrochemically exfoliated graphene (EEG) were developed using a simple hot pressing method (Figure 1b).

First, EEG–textile hybrids can be utilized both as flexible substrates as well as highly conductive gate electrodes for wearable organic field-effect transistors (OFETs). Second, the spray-coated EEG was also used for the source/drain (S/D) electrodes of the textile-based wearable OFETs, which exhibited a high mobility of 9.7 cm^2^ V^−1^ s^−1^. In our previous work, we already fabricated OFETs via spray coating with EEG and utilizing a conducting polymer hybrid for the S/D electrodes [15,16]. To reduce the roughness and sheet resistance of the EEG electrodes, subsequent coating with conducting polymer (poly(3,4-ethylenedioxythiophene)-poly(styrenesulfonate) (PEDOT:PSS)) and acid treatment were performed. However, in this work, pure EEG S/D electrodes with lower sheet resistance were developed through a simple hot pressing method without additional conducting polymer coating. Finally, the performance of OFETs was further enhanced ~1.5 times (13.8 cm^2^ V^−1^ s^−1^) by the addition of EEG nanosheets to the organic semiconductor material as a channel layer of the wearable OFETs. To the best of our knowledge, this is the first work to prepare high-performance wearable OFETs by applying graphene to all of the electrodes as well as the semiconducting channel layer.

## 2. Materials and Methods

### 2.1. Materials

Graphite foils (99.8%) were purchased from Alfa Aesar (Ward Hill, MA, USA). Ammonium sulfate ((NH_4_)_2_SO_4_, 99.5%) and N-methyl-2-pyrrolidone (NMP, 99.5%, special grade) were purchased from Samchun Chemical Company (Seoul, Korea). Poly(vinylidene fluoride-co-hexa-fluoropropylene (P(VDF-HFP), average MW ~400,000, pellets), 1-Ethyl-3-methylimidazolium bis(trifluoromethylsulfonyl)imide ([EMIM][TFSI], ≥98% HPLC), acetone (ACS reagent, ≥99.5%), toluene (anhydrous, ≥99.8%), and Cu etchant were purchased from Sigma-Aldrich (Milwaukee, WI, USA). Poly(3-hexylthiophene) (P3HT, MW = 50–70 KDa, PDI = 1.4–1.6) was purchased from Solaris Chem Inc. (Vaudreuil-Dorion, QC, Canada). The anodisc membrane filter (Φ 47 mm, 0.02 μm pore size) was purchased from Whatman (Maidstone, UK). The textile substrate was obtained by cutting a laboratory coat that was made of 100% cotton fabric.

### 2.2. Synthesis of Electrochemically Exfoliated Graphene

The EEG was prepared using a previously reported method [15]. Five graphite foils and six stainless steel (STS) plates were used as the working and counter electrodes, respectively, and 0.3 M (NH_4_)_2_SO_4_ was used as the electrolyte. The EEG was prepared by applying 10 V to each graphite electrode for 30 min, followed by vacuum filtration, washing with distilled water, and drying.

### 2.3. Fabrication of EEG/Textile Electrodes

The EEG/textile electrodes were prepared using the previously reported method [16]. Briefly, the EEG film was prepared via vacuum filtration of the EEG dispersions in NMP. Then the EEG film was placed on the flexible cotton textile substrate and hot pressed at 180 °C under a pressure of 5000 kgf cm^−^^2^ for 30 min. The EEG film was cut to a suitable size before hot pressing, and the area of the EEG electrodes on the textile substrate was adjusted to fit the transistor application.

### 2.4. Fabrication of Flexible OFET Based on EEG/Textile Electrodes with P3HT/EEG Nanocomposite Active Channel Layer

The EEG/textile electrode was used as both the substrate and gate electrode for the wearable OFET. The EEG S/D electrodes were fabricated using spray coating and hot pressing. The spray coating of EEG solution for the S/D electrodes of the flexible OFET was performed using the previously reported method [16]. After spray coating deposition, the hot pressing of the EEG S/D electrodes onto the Cu foil was carried out at 5000 kgf cm^−2^ and 200 °C for 30 min.

The ion gel layer was prepared by first codissolving P(VDF-HFP) and the ionic liquid, [EMIM][TFSI], in acetone (the weight ratio between the polymer, ionic liquid, solvent was kept to 1:4:7), and then spin coating on the washed Si wafer at 1000 rpm for 1 min. Spin-coated ion gel layers were placed in a vacuum at 70 °C for 24 h to remove the residual solvent [17]. Then, the EEG/textile gate electrode, the ion gel layer, and the EEG S/D electrodes on Cu foil were stacked together by annealing on a hot plate at 120 °C for 5 min. During this process, the EEG S/D electrodes on the Cu foil were embedded into the melted ion gel, and the EEG/textile gate electrode stuck to the ion gel at the same time. Finally, the Cu foil was etched using a Cu etching solution.

The active layer was deposited using the solution-floating method [18]. The P3HT/EEG nanocomposite solution was obtained by simple blending of the P3HT solution (in toluene, 10 mg/mL) and the EEG solution (in NMP, 1 mg/mL) in 1:1 volume ratio.

### 2.5. Characterization

The surface morphologies of the EEG film, the EEG/textile electrode, and the EEG S/D electrodes were investigated using SEM (CX-200TA, COXEM, Daejeon, Korea). Detailed surface and cross-sectional SEM images were taken from a field-emission scanning electron microscope (FE-SEM; Mira 3 LMU FEG, Tescan, Brno, Czechia). Alpha step images were obtained using a DektakXT Stylus Profiler (10th generation system, Bruker, Billerica, MA, USA) in 3-dimensional measurement, and then the root mean square (RMS) surface roughnesses (*R*_q_) of the EEG/textile electrodes and EEG S/D electrodes were also taken from the alpha step. The Raman spectra (laser wavelength: 532 nm) were obtained by a Ultima IV (Rigaku, Tokyo, Japan). The XPS was characterized on a Kratos AXIS Nova spectrometer (Shimazdu, Kyoto, Japan). The sheet resistances of the EEG films, EEG/textile electrodes, and EEG S/D electrodes were measured using a resistivity meter (FPP-40k, DASOLENG, Cheongju, Korea). The sheet resistance values from 10 different points were collected and averaged. The measurement of the electrical characteristics of the OFET devices that utilized EEG/textile electrodes was carried out at room temperature in an air conditioned environment using a MST-4000A probe station (MSTECH, Seoul, Korea) and a Keithley 2612B (Cleveland, OH, USA) source meter.

## 3. Results and Discussion

**Figure 2:**
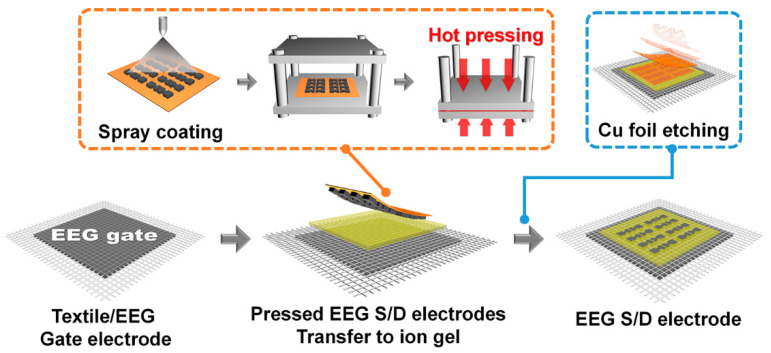
Schematic illustration of the fabrication of S/D electrodes via spray coating of EEG dispersion, followed by hot pressing.

**Figure 3:**
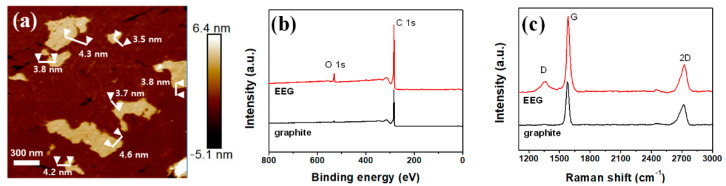
(**a**) AFM image of EEG on silicon substrate. (**b**) XPS and (**c**) Raman spectra of EEG and graphite.

Figure 2 shows the fabrication process of a flexible OFET using the EEG/textile electrode as both the substrate and gate electrode. Hereinafter, this OFET device will be abbreviated as textile-OFET. The highly conductive EEG was synthesized via electrochemical exfoliation of a natural graphite [15]. The lateral size of the EEG sheets was in the range of 3−4 μm, with a mean thickness of around 4 nm, which indicated that the EEG was composed of five layers of graphene sheets, considering that the thickness of monolayer graphene is about 0.8 nm (Figure 3a). From the high C/O ratio (16.2) of the XPS peaks and low intensity ratio of the D and G peak (*I*_D_/*I*_G_ ≈ 0.14) of Raman peaks (Figure 3b,c), we confirmed that high-quality graphene with a low defect density was successfully prepared.

**Figure 4:**
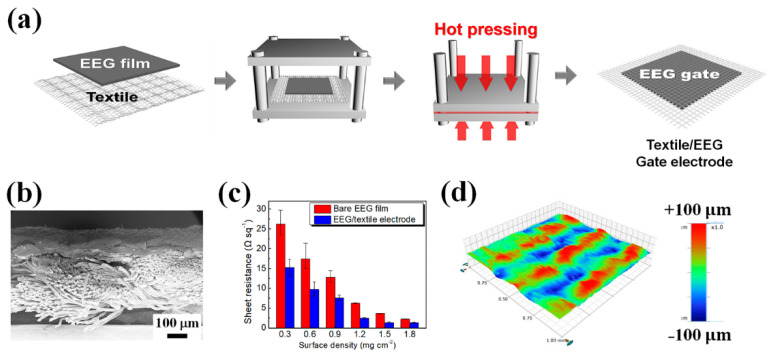
(**a**) Schematic illustration of the fabrication of EEG/textile gate electrode using hot pressing. (**b**) Cross-sectional SEM image of the EEG/textile gate electrode. (**c**) Changes in the sheet resistance of the bare EEG film and the EEG/textile gate electrode depending on the surface density of EEG films. (**d**) Surface 3D profile image of the EEG/textile gate electrode.

Figure 4a exhibits the preparation of the EEG/textile gate electrode, in which the EEG film was fabricated through vacuum filtration of the EEG aqueous solution and combined with the textile substrate through hot pressing. By varying the surface density of the EEG films, the sheet resistance and surface roughness of the EEG/textile gate electrodes was controlled. The optimized EEG/textile gate electrode, prepared by the hot pressing of the EEG film with a surface density of 1.8 mg cm^−2^ to the textile, had a low sheet resistance of 1.30 Ω sq^−1^ (Figure 4c) and a smooth surface with an RMS roughness of 6.88 µm (Figure 4d). It was used as the gate electrode of the textile-OFET to reduce the contact resistance between the device interfaces.

**Figure 5:**
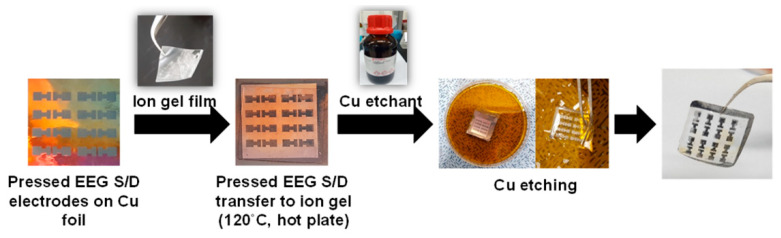
Photo images of the manufacturing process of the hot-pressed EEG S/D electrodes via transferring to the ion gel dielectric layer and Cu foil etching.

To obtain the flexibility of the textile-OFET, an ion gel was applied to the dielectric layer (Figure 5). Although it has been pointed out that the on-current of the OFET is exaggerated due to the higher capacitance value of the ion gel (9.0 mF, 11 μm thickness) compared to that of the conventional SiO_2_ (11.5 nF, 300 nm thickness), ion gel is widely used as both a flexible dielectric layer and robust transporter of the electrodes as well as a channel material for the fabrication of flexible OFET devices [19]. In addition, the S/D electrodes were fabricated as flexible electrodes with a low sheet resistance using the spray coating method of the EEG dispersion followed by hot pressing. The detailed manufacturing process of the S/D electrodes consists of the following four steps: (i) spray coating the EEG dispersion onto Cu foil, (ii) hot pressing, (iii) transferring to the ion gel dielectric layer, and (iv) Cu foil etching.

**Figure 6:**
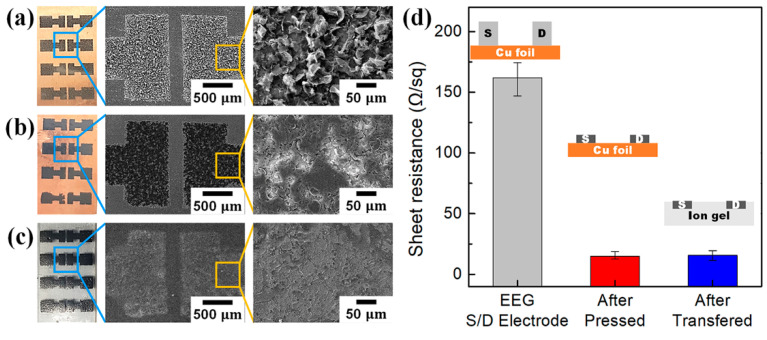
Photo and SEM images of EEG S/D electrodes after (**a**) spray coating onto Cu foil, (**b**) hot pressing, (**c**) transferring to ion gel dielectric layer, and Cu foil etching. (**d**) Changes in the sheet resistance of EEG S/D electrodes after each step.

**Figure 7:**
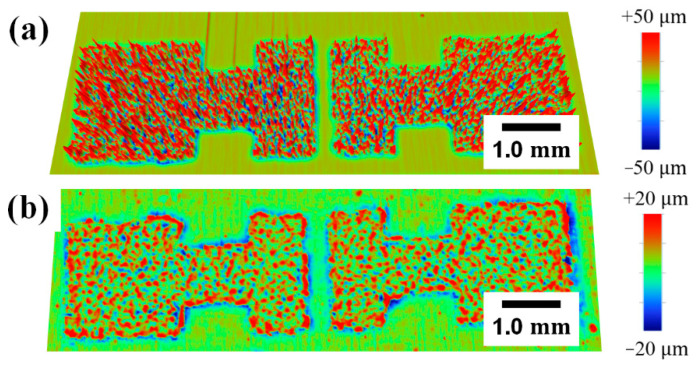
Surface 3D profile images of EEG S/D electrodes after (**a**) spray coating onto Cu foil and (**b**) hot pressing.

Figure 6 shows the variations in the surface morphology and sheet resistance of the EEG S/D electrodes in each step. The surface of the EEG S/D electrodes formed by the spray coating onto the Cu foil was very rough, like an EEG film surface after vacuum filtration, and the EEG sheets were oriented in random directions (Figure 6a). However, after the hot pressing, the surface of EEG S/D electrodes became very smooth (flat surface), and the EEG sheets were mostly oriented in a direction horizontal to the Cu foil surface (Figure 6b). An analysis of the RMS roughness using the alpha step confirmed that the roughness of the spray-coated EEG S/D electrodes decreased sharply to an approximately 1/15 level, from 5.12 µm to 0.36 µm, after hot pressing (Figure 7). Furthermore, even after the EEG S/D electrodes were transferred to an ion gel dielectric layer and then underwent Cu foil etching, the microstructure of the EEG S/D electrodes was well maintained in the dielectric layer, without any damage (Figure 6c).

As shown in the changes of the sheet resistance of the EEG S/D electrodes in Figure 6d, after the hot pressing the sheet resistance of EEG S/D electrodes dropped sharply to an approximately 1/10 level, from 162 Ω sq^−1^ to 14.8 Ω sq^−1^. The reason for the decrease in the RMS roughness and the sheet resistance of the EEG S/D electrodes is that the thickly spray-coated graphene sheets in random directions on the Cu foil surface were oriented horizontally by the hot pressing, and the conductive pathway was improved by thermal reduction. The hot-pressed EEG S/D electrodes did not show a significant change in sheet resistance (15.9 Ω sq^−1^) even after being transferred to the dielectric layer (Figure 6d). The transferred EEG S/D electrodes were not simply loaded on the surface of the ion gel-based dielectric layer, but embedded inside the ion gel that was melted by heat treatment, as shown in the rightmost part of the graph in Figure 6d. As a result, the microstructure of the EEG sheets constituting the S/D electrodes rarely changed, and the electrical properties of the electrodes were well-maintained even after repeated bending by external forces because the elastic ion gel supported the EEG S/D electrodes. Similarly, Chortos et al. fabricated a CNT device in an embedded form inside an elastic polymer and found that the properties of the device were maintained even after repeated shape changes caused by external forces [20].

**Figure 8:**
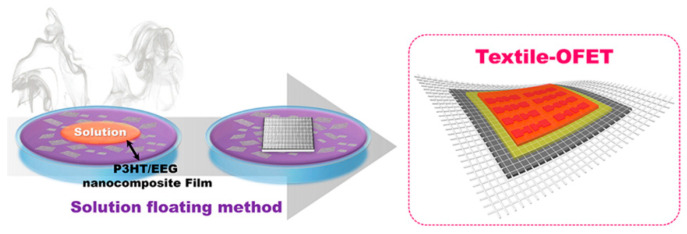
Schematic illustration of solution floating method for depositing the P3HT/EEG nanocomposite film onto textile-OFET.

The solution-floating method (SFM) was used for depositing the active channel layer, which was the last step of fabricating the textile-OFET, as illustrated in Figure 8. Until now, many deposition methods, such as spin coating, inkjet printing, and the roll-to-roll method, have been used to apply solution-processable organic semiconducting channel materials onto rigid and flat organic devices. However, these methods are not suitable for application to wearable devices with flexible and curved surfaces. The SFM is a highly efficient deposition method, not only in terms of forming the well-crystalline polymer semiconductor channel in wearable devices, but also in terms of enhancing electrical properties and easily imparting new functions of the polymer semiconductor channel by combining the nanomaterials [21].

We completed the textile-OFET by applying the P3HT/EEG nanocomposite film using the SFM, which was optimized in a previous study [22] where the OFET devices fabricated using P3HT/EEG nanocomposite films with a mass ratio of 10:1 exhibited a nearly twice as high field-effect mobility compared to the OFETs developed using pristine P3HT, as well as showing an order of magnitude amplification of the OFET on–off ratio. Consequently, EEG was applied to all of the components of the textile-OFET, from the gate electrode and S/D electrodes to the active channel material. To the best of our knowledge, this is the first report to fabricate wearable OFETs by applying graphene to all electrodes (gate and S/D electrodes) as well as the semiconducting channel layer.

**Figure 9:**
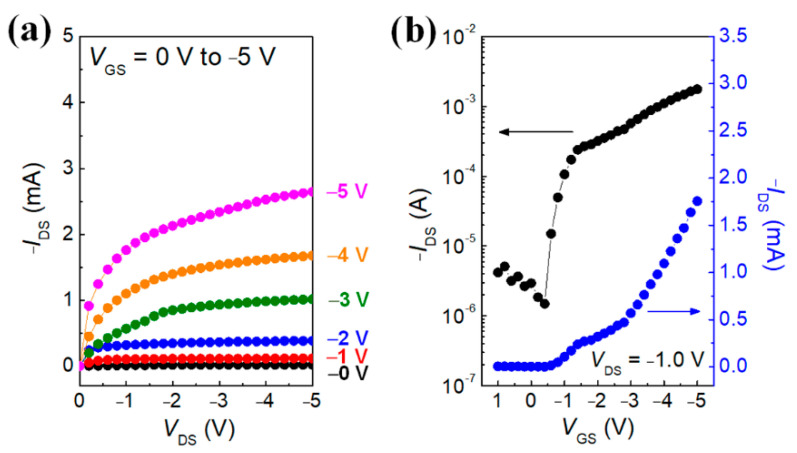
(**a**) Output and (**b**) transfer characteristics of textile-OFET using pure P3HT as an active channel material.

Figure 9 shows the electrical characteristics of the textile-OFET developed using the pure P3HT active channel layer. Figure 9a shows good output characteristics using gate modulation. Furthermore, the field-effect mobility (*µ*_FET_) of the textile-OFET can be obtained through the linear regime of the transfer curve (Figure 9b) by using the following equation:

$$I_{\mathrm{DS}}=\frac{W}{L}\times C_{i}\times \mu_{\mathrm{FET}}\times V_{\mathrm{DS}}\times \left(V_{\mathrm{GS}}-V_{\mathrm{Th}}\right)$$

where *I*_DS_ is the drain current, *W* is the channel width (2000 μm), *L* is the length (300 μm), $C_{i}$ is the capacitance of ion gel (9.0 μF, corresponding to a thickness of 11 μm), *V*_DS_ is the drain voltage, *V*_GS_ is the applied gate voltage, and *V*_Th_ is the threshold voltage, respectively. The *μ*_FET_ of the textile-OFET developed using the P3HT active channel layer, calculated from the transfer curve in Figure 9b, is 9.7 cm^2^ V^−1^ s^−1^, which is a much higher value than those reported in other P3HT-based transistors gated with conventional silicone oxide dielectrics (0.1–0.01 cm^2^ V^−1^ s^−1^). However, it has already been reported that this phenomenon occurs in many research groups for the following reason: the ions permeate from the ion-gel dielectric to the P3HT active channel, which fills the carrier traps and acts as a dopant in the P3HT channel layer [23].

**Figure 10:**
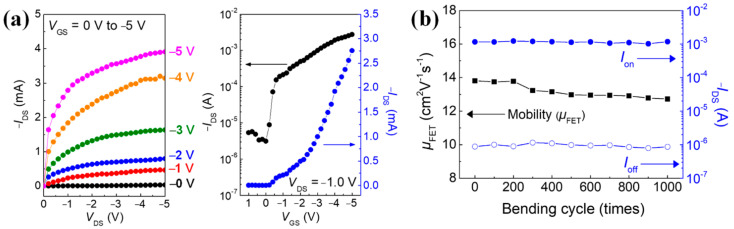
(**a**) Output and transfer characteristics of the textile-OFET using P3HT/EEG nanocomposite as an active channel material. (**b**) Changes in the field-effect mobility and on and off currents of the textile-OFET developed using P3HT/EEG nanocomposite as dependent on bending cycles (bending radius = 1.5 cm).

Figure 10 shows the electrical characteristics of the textile-OFET developed using on the P3HT/EEG nanocomposite (mass ratio of 10:1). The *μ*_FET_ of the textile-OFET developed using the P3HT/EEG nanocomposite, as calculated from the transfer curve in Figure 10a, is 13.8 cm^2^ V^−1^ s^−1^, which is ~1.5 times amplified compared to the textile-OFET developed using a pure P3HT active channel layer (Table 1). Therefore, the electrical characteristic of amplification through EEG hybridization appears to be beneficial for the textile-OFET, as with the result of the previous research [22]. Hence, it can be concluded that the deposition of the active channel layer developed using the P3HT/EEG nanocomposite was completed successfully via the SFM, and the device developed using EEG electrodes (spray-coated EEG S/D and hot pressed EEG/textile gate electrode) operated well.

The graph in Figure 10b shows the measurements of the *μ*_FET_ and the on and off currents during 1000 cyclic bending tests (bending radius = 1.5 cm) that were conducted to prove the flexibility of the textile-OFET. The *μ*_FET_ of the textile-OFET decreased from 13.8 to 12.7 cm^2^ V^−1^ s^−1^ after 200 bending cycles. This reduction of mobility may be due to the increased contact resistance between the interfaces of devices caused by repeated bending strains. However, it is not a significant decrease, and confirms that the properties of the textile-OFET were not deteriorated [24]. We also found that the on and off currents of the textile-OFET showed almost no change during the 1000 bending cycles. The reason for this is that all the components of the textile-OFET—the EEG/textile gate electrode, the ion gel dielectric layer, the EEG S/D electrodes, and the P3HT/EEG nanocomposite active layer—have flexible and durable properties; additionally, the EEG/textile gate electrode also serves as a flexible substrate that firmly supports the components of the textile-OFET.

## 4. Conclusions

The EEG/textile electrode was prepared by hot pressing the EEG film to the cotton textile, and it exhibited a low sheet resistance of *R*_s_ of 1.3 Ω sq^−1^ and a smooth surface with an RMS roughness of 6.88 µm. OFETs were fabricated using the EEG/textile electrode as both the gate electrode and the flexible substrate to demonstrate their application in wearable electronic devices. In addition to the EEG/textile electrode, the S/D electrodes were prepared using a spray coating of the EEG dispersion followed by hot pressing. After the hot pressing, the surface of EEG S/D electrodes became very smooth and the roughness decreased from 5.12 µm to 0.36 µm. Furthermore, the sheet resistance of the EEG S/D electrodes dropped sharply from 162 Ω sq^−1^ to 14.8 Ω sq^−1^. Finally, the wearable OFET devices were successfully prepared by applying the P3HT/EEG nanocomposite film as an active channel layer using the SFM, that is, creating the first wearable electronic devices by applying the graphene to all electrodes as well as the semiconducting channel layer. The field effect mobility of the OFET developed using the P3HT/EEG nanocomposite was 13.8 cm^2^ V^−1^ s^−1^, which is ~1.5 times amplified compared to the textile-OFET developed using pristine P3HT film (*μ*_FET_ = 9.7 cm^2^ V^−1^ s^−1^). In addition, the textile-OFET device exhibited stable electrical performances during the 1000 bending cycles, owing to the flexible and durable properties of all of its components. Consequently, a fabrication method for wearable transistors based on textiles and hot-pressed graphene electrodes is expected to be applied to the next generation of wearable electronic devices.

## Figures and Tables

**Table 1:** Average field-effect mobilities, on–off current ratios, and threshold voltages for textile-OFETs based on P3HT and P3HT/EEG nanocomposite as active channel materials.

Active Channel Material	*μ*_FET_ (cm^2^·V^−1^·s^−1^)	*I*_on_/*I*_off_	*V*_Th_ (V)
P3HT	9.7	~10^3^	−2.00
P3HT/EEG nanocomposite	13.8	~10^3^	−1.41

## Data Availability

Not applicable.
